# Introducing a New Smartphone Applied Semen Analyzer, SpermCell™: A Cross-Sectional Validation Study with a Comparative Analysis and a Mini Patient Questionnaire on a Large Sample Cohort

**DOI:** 10.3390/diagnostics14070689

**Published:** 2024-03-25

**Authors:** Muhammet Murat Dincer, Serhat Yentur, Aykut Colakerol, Gokhan Cil, Ramazan Omer Yazar, Engin Kandirali, Atilla Semercioz, Ahmet Yaser Muslumanoglu, Mustafa Zafer Temiz

**Affiliations:** Department of Urology, Bagcilar Training and Research Hospital, Istanbul 34200, Turkeyaykutcolakerol@hotmail.com (A.C.); dromeryazar@gmail.com (R.O.Y.); ymuslumanoglu56@hotmail.com (A.Y.M.); dr_mustafazafertemiz@hotmail.com (M.Z.T.)

**Keywords:** medical device, point-of-care testing, smartphone, semen analysis, validation study

## Abstract

(1) Background: Standard semen analysis methods may exhibit variability between observers and/or human error; therefore, additional methods are needed to overcome these handicaps. We aimed to present a new smartphone-applied semen analyzer, Sperm Cell™, investigate its diagnostic efficacy by comparing it with the standard analysis method, and determine its user-friendly nature. (2) Methods: A cross-sectional study was conducted on a large sample cohort, including 102 men. Three semen analyses were performed for each semen sample. The first employed the standard manual method, whereas the others were smartphone-based analyses performed by technicians and patients. We compared major semen parameters between the three semen analyses. The user-friendly nature of the analyzer was also evaluated with a mini-questionnaire completed by the participants. (3) Results: The determined median sperm count, motile sperm count, and percentage of motile sperms, on standard manual semen analysis, were 50.00 × 10^6^/mL (0–160 × 10^6^/mL), 23.94 × 10^6^/mL (0–108 × 10^6^/mL) and 50.00% (0–73.00%), respectively. Median sperm count and motile sperm count were 50.52 × 10^6^/mL (<1–150 × 10^6^/mL) vs. 55.77 × 10^6^/mL (<1–160 × 10^6^/mL) and 23.34 × 10^6^/mL (0–105 × 10^6^/mL) vs. 23.53 × 10^6^/mL (0–104 × 10^6^/mL) for SpermCell™-based semen analysis performed by a technician and patients themselves, respectively. The percentages of motile sperms were 47.40% (0–67.00%) vs. 47.61% (0–80.20%), respectively. All the parameters were statistically similar between the three semen analysis methods (*p* > 0.05 for each). The SpermCell™ analysis results were correlated with the standard manual method with up to 0.85 correlation coefficients. Moreover, substantial diagnostic accuracy, sensitivity and specificity were obtained in determining the oligospermia and asthenozoospermia via the device-based analyses performed by technician and patients. The mini-questionnaire results revealed that the analyzer is useful. (4) Conclusions: The novel smartphone-applied semen analyzer is a helpful tool with acceptable diagnostic accuracy in determining the major semen parameters. It can be used as an efficient at-home point-of-care testing method in the initial assessment of couples with infertility concerns.

## 1. Introduction

The investigation of semen samples is a mandatory diagnostic approach in managing male infertility [1]. It provides valuable information about the possible causes of infertility and could help institute appropriate therapy [2]. Semen analysis originated in the 1670s, with the first observation of human sperm cells using the microscope created by Anton Van Leeuwenhoek [3]. Then, for the first time in years, Capanna and Spallanzani [4] documented the fertilizing capacity of sperm in 1771, and numerous authors continued to investigate human semen [5]. As a result, several methods that various investigators have suggested have been used. Nevertheless, most of them exhibited methodological variabilities and encountered limitations not only in terms of semen analysis technique, but also lacked sufficient reliable data [1]. In an attempt to reduce variability, one method of semen analysis has been standardized by the World Health Organization (WHO) since the 1970s, with a detailed manual for producing, editing, updating, and disseminating a semen analysis [6]. Although, today, the analysis of semen samples can be described as a standardized clinical investigation tool, it is a subjective procedure that is typically performed manually by a human operator. Therefore, a high level of experience is required to minimize the subjectivity during the microscopic assessment of the sperm count, motility, and morphology. But still, the results may exhibit variability between observers, and/or human error may affect the analysis results, acting as an obstacle to accurate semen analyses [7,8].

Several additional methods for the investigation of semen samples have been propounded to avoid human subjectivity and potential errors in the results [8]. Most methods include computer-assisted or smartphone application-based analytical tools for investigating semen parameters. However, computer-assisted methods are expensive and sometimes inefficient in terms of calibration [8]. On the other hand, in some cases, smartphone-based methods involve potential user error risks due to their design or technical aspects. Moreover, the lack of adequate validations and quality control of those methods may lead to false-negative results, which could delay the diagnosis or treatment process [9]. In this regard, any new method must be inexpensive, easily accessible, user-friendly and, first and foremost, correctly validated in terms of its user-friendly nature and accuracy. Herein, we present a new smartphone-applied semen analyzer, SpermCell™ (HİLLAL Biotech. Bioeng. Inc., Izmir, Turkey), along with an investigation of its diagnostic efficacy in a large sample cohort of real-life patients, by comparing it with the standard manual semen analysis method. On top of that, as a novel aspect of the current work, we investigated the user-friendly nature of the device using a mini-patient questionnaire and comparing the results obtained from patients’ and laboratory staff analyses.

## 2. Materials and Methods

A cross-sectional validation study with a comparative analysis and a mini patient questionnaire was conducted on a large sample cohort, including 102 men, between April 2022 and June 2022. The study protocol was reviewed and approved by the Istanbul Biruni University Review Board (Approval No. 2020.05.1.08.040). Informed consent was obtained from all subjects when they were enrolled and the principles outlined in the Declaration of Helsinki were followed. After approval, the study was started by introducing and evaluating a new smartphone-applied semen analyzer for point-of-care semen analysis tests.

The study included men who applied to our urology clinic with complaints of male infertility and/or scrotal pain due to clinical varicocele. Exclusion criteria were having a history of previous device usage and physical or neurological disabilities. Three semen analyses were performed for each unprocessed semen sample. The first was a standard manual method, whereas the others were smartphone-based analyses performed by technicians and patients. We evaluated and compared the sperm counts and motilities in the three semen analyses. The user-friendliness of the analyzer was also evaluated with a mini-questionnaire, with two questions (described in the results section) completed by the participants.

Semen samples were obtained by masturbation, and all of the ejaculate was collected into a sterile plastic container after an asexual abstinence period of two to five days. Three semen analyses with single measurements were performed synchronously for each semen sample obtained from a man. The first was the standard manual method performed according to the World Health Organization Laboratory Manual for the Examination and Processing of Human Semen (Sixth Edition) [10]. The second and latter ones were the smartphone-applied semen analyzer and SpermCell^TM^-based semen analyses performed by two experienced laboratory technicians and patients, respectively. Our laboratory technicians were trained by manufacturer staff and performed ten analyses using SpermCell™ with the aim of training and developing experience before the study protocol. An instruction-to-use form and a product information form were presented to the patients, who were completely unfamiliar with the device. No further verbal or visual explanation or demonstration was provided so as to evaluate the user-friendly nature of the device correctly. The manufacturer’s staff were not present during the device’s usability testing.

A blind protocol was adopted during the study, and the patients’ identifiers, including their names, were hidden from the laboratory technicians. We used random numbers from 1 to 210 to identify the semen samples investigated by the laboratory technician via manual and smartphone-applied analysis methods. After completing the smartphone-applied semen analysis, the laboratory technicians sent the system outputs to the analysis report center with the identification number for each analysis via a specific smartphone application working with an e-mail account (created for the study). On the other hand, patient-derived smartphone-applied semen analysis outputs and personal identifiers were sent by patients via a specific smartphone application working with their e-mail accounts.

We evaluated the main semen parameters, including volume, pH, sperm counts, motility, percentage of normal morphology, and seminal leukocytes, with the manual method. However, sperm counts and motility were the only parameters for the SpermCellTM-based semen analysis. All analyses were performed on undiluted, unprocessed, and fresh semen samples. Sample liquefaction was performed at room temperature for 20–30 min before analysis.

**Figure 1:**
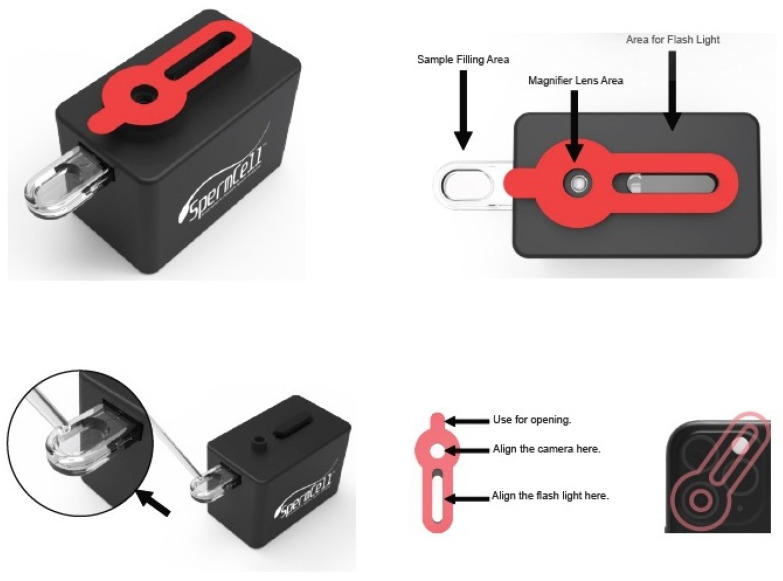
SpermCell^TM^ smartphone-applied semen analyzer.

**Figure 2:**
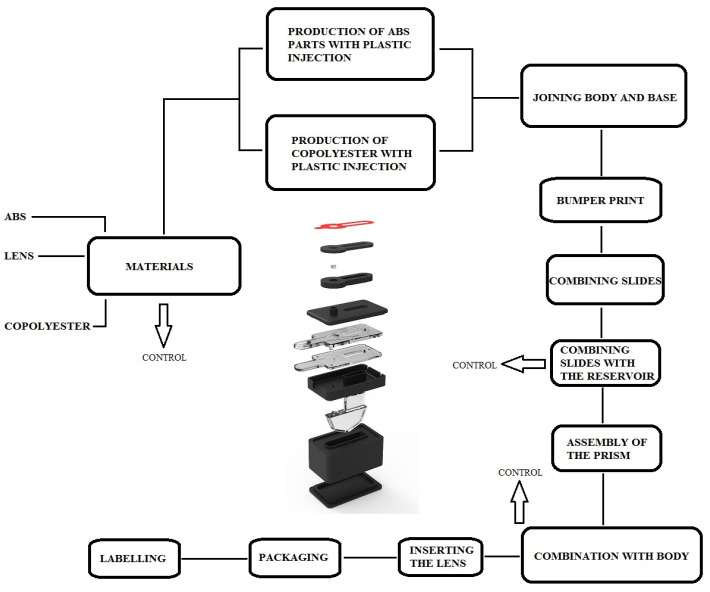
The schematic summary of the SpermCell^TM^’s fabrication procedure.

**Figure 3:**
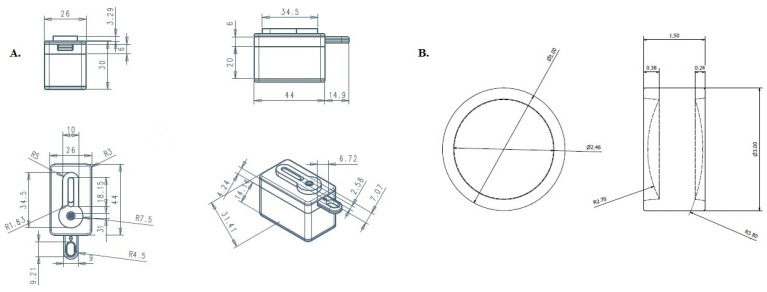
The engineering drawing of the SpermCell^TM^. (**A**) Main body, (**B**) The lens.

The components of SpermCell™ are packaged in a kit consisting of the SpermCell™ main body, a smartphone connector, a sample collector, a sampling pipette, a camera cleaning cloth, and instruction for use (Figure 1). The schematic summary of the fabrication procedure of the device is provided in Figure 2. The device is disposable and it has a measurement range of between 1 million and 250 million sperm cell counts. SpermCell™ has a lens with a 2.46 mm in diameter and two types of magnification: optical and digital. The optical magnification, determined by the distance between the aspherical lens and the area to be viewed, is approximately 300×, and remains the same for all phones. Additionally, a 1.5× digital zoom can be applied during video recording on the phone, providing an overall magnification of around 450×. It is important to note that this magnification may vary depending on the phone model. Detailed information about the product dimensions, described via engineering drawings of the Spermcell™’s main body and lens, is given in Figure 3.

Before testing, the specific smartphone application of SpermCell™ was downloaded from the Play Store of the Android Operating System or the Apple App Store of the iPhone Operating System (IOS). The study was conducted on the personal smartphones of the patients and the Galaxy S 10 smartphones (Samsung Electronics Co., Ltd., Suwon, Korea) of our laboratory technicians.

The testing procedure started by loading one drop of sample taken with a pipette from the kit into the sample area of the sample collector on the main body. Then, the main body was left to stand until the dropped sample had disappeared from the naked eye. Subsequently, the lens and flashlight parts of the main body were attached to the main camera and flashlight of the smartphone for capturing the image of the sperm cells on the high-definition screen. After automatically displaying a video image of the sample, the video output is sent to the analysis report center through the application. The analysis results are automatically sent back to the user’s phone.

Statistical analysis was performed using the IBM SPSS Statistics version 22.0 statistic software package (IBM SPSS Inc., Chicago, IL, USA). Data distributions and tests of normality were evaluated with the Kolmogorov–Smirnov test. Descriptive statistic methods (mean ± standard deviation and median ± interquartile range) were used to evaluate the data. We compared the parametric data that were not normally distributed between the three groups using the Kruskal–Wallis test. The chi-square test was also used to compare the nonparametric categorical variables. Correlation analyses were performed using Spearman’s correlation test. Diagnostic sensitivity and specificity were also determined with a 2 × 2 sensitivity and specificity table. Differences were significant at *p* < 0.05 and the 95% confidence interval.

## 3. Results

The mean age was 31.28 ± 6.63 years in our population. The indications of semen analysis were male infertility in 71 patients (69.60%) and varicocele in the 31 remaining patients (31.40%). The mean ages were 31.61 ± 7.89 and 31.14 ± 6.05 years in the male infertility and varicocele patient groups, respectively (*p* = 0.12).

The determined median sperm count, motile sperm count, and percentage of motile sperms, measured via standard manual semen analyses, were 50.00 × 10^6^/mL (0–160 × 10^6^/mL), 23.94 × 10^6^/mL (0–108 × 10^6^/mL) and 50.00% (0–73.00%), respectively. The median sperm count and motile sperm count were 50.52 × 10^6^/mL (<1–150 × 10^6^/mL) vs. 55.77 × 10^6^/mL (<1–160 × 10^6^/mL) and 23.34 × 10^6^/mL (0–105 × 10^6^/mL) vs. 23.53 × 10^6^/mL (0–104 × 10^6^/mL) according to the SpermCell™-based semen analysis performed by technicians and the patients themselves, respectively. The percentages of motile sperms were 47.40% (0–67.00%) vs. 47.61% (0–80.20%), respectively. All the parameters were statistically similar between the three semen analysis methods (*p* > 0.05 for each) (Table 1). Two videos captured by the device exhibiting motile and non-motile sperms may be seen in the Supplementary Videos S1 and S2.

**Figure 4:**
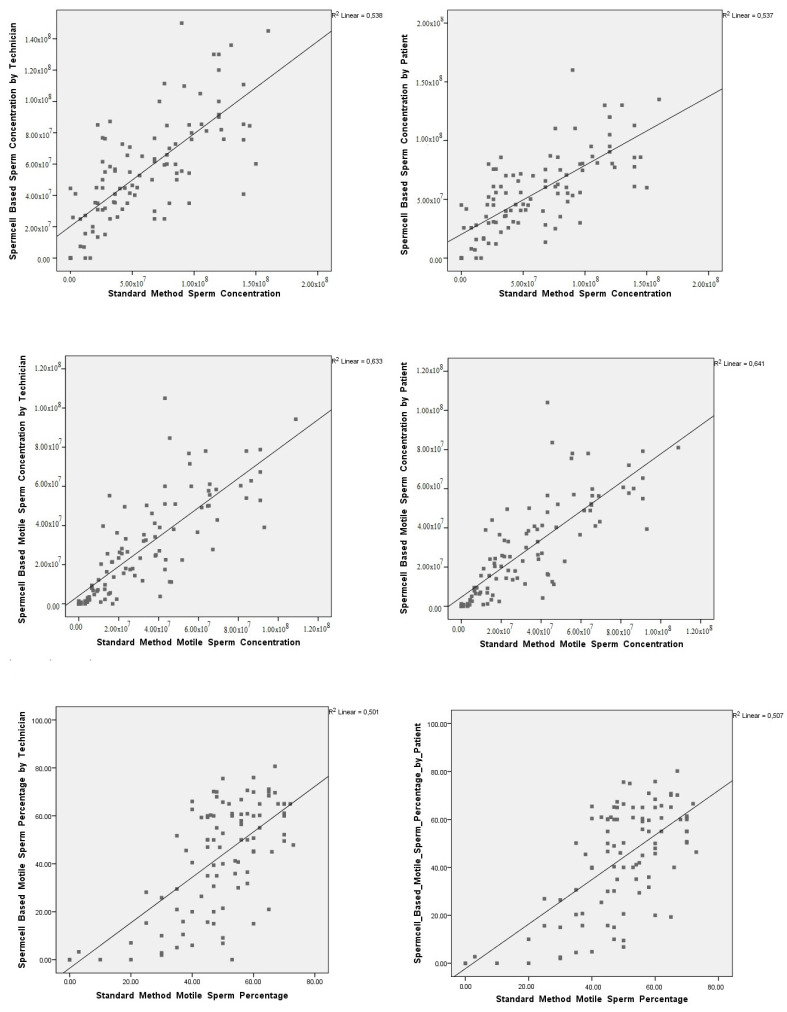
Correlation graphs for the parameters in the different semen analysis methods.

Comparisons between values of sperm concentration, total motile sperm count, and motile sperm percentage obtained using the standard manual method with those obtained using the SpermCell^TM^-based method by technicians and patients themselves resulted in a statistically significant correlation, with correlation coefficients of 0.742 and 0.748, 0.848 and 0. 858, and 0.629 and 0.616, respectively (Figure 4).

The SpermCell™-based semen analysis performed by the technicians yielded false-positive and false-negative oligospermia (according to the standard manual of semen analysis) in two and six patients, respectively. Therefore, the diagnostic accuracy was 92.15% (94 (exactly diagnosed cases)/102 (total cases): 0.9215) in relation to determining oligospermia. False-positive and -negative oligospermia diagnoses were yielded in four and six patients, respectively, via SpermCell™-based semen analyses performed by patients. As a result, the diagnostic accuracy was determined to be 90.19% (92 (exactly diagnosed cases)/102 (total cases): 0.9019). In determining asthenozoospermia, false-positive results were yielded in 20 and 18 patients from tests administered by technicians and patients, respectively. On the other hand, false-negative diagnoses were determined in two patients in each group. The diagnostic accuracies were 78.43% and 80.39%, respectively (80 (exactly diagnosed cases)/102 (total cases): 0.7843 and 82 (exactly diagnosed cases)/102 (total cases): 0.8039, respectively). The diagnostic sensitivity and specificity of the SpermCell^TM^-based semen analysis methods were 60.00% and 97.70% and 60.00% and 95.40% for oligospermia, and were about 91.00% and 77.00% for asthenozoospermia, respectively (Table 2, Table 3, Table 4 and Table 5).

**Figure 5:**
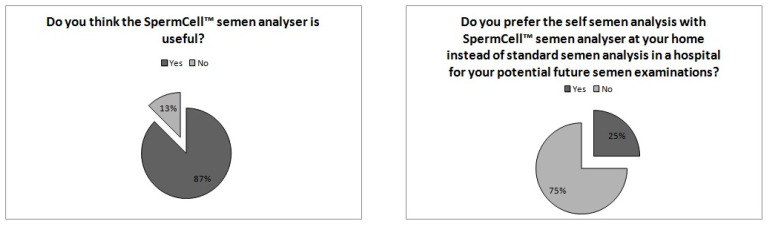
Results of the patient mini-questionnaire on pie charts.

The mini-questionnaire results reveal that 87% of the participants answered “Do you think the SpermCell^TM^ semen analyzer is useful?” with a “yes”. However, only 25% of them answered the question ‘‘Do you prefer the self-semen analysis with the SpermCell^TM^ semen analyzer at home instead of standard semen analysis in a hospital for your potential future semen examinations?’’ with ‘‘no’’ (Figure 5).

## 4. Discussion

Fertility refers to the capacity to establish a clinical pregnancy. However, some couples cannot reach a clinical pregnancy because of numerous factors. The diagnostic term infertility describes this inability when it appears after regular unprotected sexual intercourse for one year [11,12]. While infertility diagnosis is such a simple process that it can be reached via a self-diagnosis for couples with fertility issues, investigating causative factors that may originate from male, female, or combined male–female factors is sometimes more complex [12]. The first and simplest investigative tool involves the analysis of semen samples obtained from male partners of infertile couples [13].

Nevertheless, men are more hesitant to seek professional medical evaluations than their female partners [14,15]. The primary cause of men’s hesitancy is their knowledge of the stressful and embarrassing nature of producing semen samples in a hospital setting [16]. In current urological practice, semen analysis is still performed by a professional practitioner after obtaining the samples in a medical facility, and this is an essential drawback of semen analysis. On the other hand, variability between observers and/or human error can problematize accurate results during semen analysis [7,8].

To overcome these faults, novel technical developments have been integrated into the test. For instance, the computer-assisted semen analysis (CASA) method obtains accurate and objective results by eradicating the human factor [17]. CASA has shown high consistency, accuracy, and repeatability during the semen analysis. Many studies have reported on the CASA method as a valid alternative to the standard semen analysis method for sperm concentration and motility [8]. Despite their usefulness, commercial CASA methods are expensive [8,18] and require significant technical skill [19]. Moreover, they cannot reduce men’s hesitancy because they are still technically based in a clinic or hospital setting.

To reduce cost and enable usefulness, several studies have recently introduced a more practical semen analysis method using mobile phone technology. These commercially available tests aim to screen male fertility parameters at home as a point-of-care method [9,20,21]. With these test kits, it is possible to determine several semen parameters, such as sperm count, sperm motility, and motile sperm count, but not sperm cell morphology. However, many of them only allow users to test sperm concentration, which is only one aspect of the semen analysis process employed to assess fertility potential. Only a few kits allow for the simultaneous investigation of sperm concentration, motile sperm count, and sperm motility [9]. The most significant aspect limiting the use of smartphone-applied semen analysis technology may be this lack of sperm cell morphology evaluation. However, some clinical studies have reported that abnormal sperm morphology may not have a negative impact on pregnancy following natural conception or intrauterine insemination [22]. It has been shown that the total motile sperm count is more predictive of fecundity than other semen parameters, including morphology [23,24]. In this regard, an accurate smartphone-applied semen analysis method that can describe sperm counts and motility may be valuable and helpful in andrology clinical practice. In particular, this method may offer the advantage of simple practicality as well. This point is an essential aspect of semen analysis, because many men struggle with providing a semen sample in a laboratory setting [25]. With the smartphone-aided semen analysis method, a man can obtain his semen analysis report following at-home self-testing. This is thus a convenient, rapid, and cost-effective way to identify men with subfertility without personal stress or hesitancy [9]. To incorporate any smartphone-applied semen analysis kit into routine clinical usage, it must be validated in terms of accuracy, efficacy, usefulness, and applicability. However, studies introducing a new smartphone-applied semen analysis program have validated their methods primarily in terms of accuracy by comparing the results obtained from the devices with those obtained from standard manual semen analyses [9,26]. However, the majority of these accuracy tests were conducted on a limited number of semen samples and/or in healthy men [27,28]. In the present study, we validated the new device in terms of accuracy by comparing standard manual semen analysis methods in a large number of real-life patients, with the additional assessment of usefulness and applicability. We found that SpermCell™ yielded similar findings to the standard manual method. All the investigated semen parameters were statistically similar between the reports of SpermCell™ and those of standard manual semen analyses. Moreover, the sperm count, sperm motility, and total motile sperm counts determined via SpermCell™ analysis correlated with those provided by the standard manual method, as has been previously reported for other smartphone-based devices [19,28]. The diagnostic accuracies were 90.19% and 92.15% for the technician- and patient-based analyses, respectively, in relation to determining oligospermia. In relation to diagnosing asthenozoospermia, they were 78.43% and 80.39%, respectively. As a result, the diagnostic accuracy of SpermCell™ is comparable with those of other smarphone-based devices. In the literature, diagnostic accuracies in detecting abnormal semen samples based on sperm concentration were reported as 95.73% and 96.77% for trained and untrained users of such a device by Kanakasabapathy et al. [19], in 2017. These results were 96.77% and 97.31% in relation to detecting asthenozoospermia by trained and untrained users, respectively. Further, in 2018, the accuracy of another device was reported as 97.80% for motile sperm count in 144 semen samples from 24 healthy men (Agarwal et al.) [27]. On the other hand, for one of the most recently developed smartphone-based semen analysis devices, the overall accuracy was reported as 84.6% in detecting sperm motility (2021) [29]. The diagnostic sensitivity and specificity of SpermCell^TM^-based semen analyses were also considerable when using them to determine oligospermia and asthenozoospermia. The test achieved a specificity of 95.40% for the self-determination of oligospermia, whereas it had a sensitivity of 60%. This means that if the test result is positive (the sperm concentration is or more than 15 × 10^6^/mL) for a man, we can infer that he is almost wholly healthy regarding semen concentration. On the contrary, if the test result is negative (sperm concentration lower than 15 × 10^6^/mL), he might be oligospermic, with a possibility of 60%, and, therefore, he must consult a urologist or andrologist. In this regard, with the use of SpermCell^TM^ as a point-of-care home test, unnecessary hospital admissions and costs may be reduced. The sensitivity and specificity values reached in relation to determining oligospermia in the present study seem to be better than those for most of the other devices previously described in the literature. For instance, Kobori et al. [30] reported an overall sensitivity of 87.50% and overall specificity of 90.90% for their novel device applied in determining the sperm count for 50 semen samples obtained from volunteers. According to their results, the sensitivity varied between 75.00% and 90.90%, and the specificity varied between 87.80% and 90.90%, with three different smartphone models. Similarly, SpermCell^TM^ also provided us with favorable sensitivity and specificity results (91.13% and 77.21%) when used in the self-diagnosis of asthenozoospermia via patient analysis. These results are also consistent with others in the literature, such as the findings of 98.04% and 93.94% related to an untrained user performing analysis with a similar device, as derived by Kanakasabapathy et al. [19]. On the other hand, our diagnostic accuracy in relation to asthenozoospermia was better than the findings of Park et al. [29], who described sensitivity and specificity values for their smartphone-based analyzer when assessing sperm motility of 92.6% and 66.7%, respectively. Further, we evaluated the efficacy and applicability of SpermCell™, and found that test results obtained from lay patients were statistically similar to those obtained from trained laboratory staff. Finally, after evaluating the device’s ease of use with a mini patient questionnaire, we deduced the useful nature of the analyzer. The great majority of the patients (87%) reported that the SpermCell^TM^ semen analyzer was useful. However, only 25% said they would prefer semen self-analysis using SpermCell™ at home instead of in a hospital for future semen examinations. We think that the social conceptions of the professional support provided during medical treatments and unfamiliarity with the novel point-of-care technology in our country are the major causes of this discordance in the mini questionnaire. On the other hand, in a recent relevant study [31] that investigated the capacity and accuracy of a new smartphone-based semen analysis platform in 2022, only 11.10% of the participants regarded the results of the smartphone-based analysis as strongly reliable. In our opinion, the conceptions regarding professional support provided during medical treatment are similar worldwide.

The major limitation of our research was the use of SpermCell™ by the participants as a semen self-analysis platform at the hospital instead of at home. Additionally, differences in the camera technology and settings of the patients’ smartphones might have affected the results. Technically, sensor performance may affect the device’s accuracy. However, this is more dependent on user’s habits than on in situ characteristics. So, usage errors significantly affect the performance of the product. For example, the improper positioning of the lens and sensor on the camera may lead to only capturing video images in a small portion of the sample, and this could affect the results. To overcome this issue, additional comparisons between the technician’s and patients’ SpermCell™ analysis results were performed in the current study.

However, our study also has important strengths, such as the performance of simultaneous semen analyses on the same semen samples, the involvement of real-life patients, the considerable sample size and the use of a mini-questionnaire survey.

## 5. Conclusions

In the near future, technical advancements and innovations such as microfluidic chips and advanced micro-imaging systems [32,33,34] may impart the ability to quantitatively and rapidly evaluate all semen parameters, including seminal reactive oxygen species, sperm morphology and sperm DNA integrity, without the major disadvantages shown by standard semen analysis, such as the high costs, the requirement of skilled technicians, and men’s hesitancy. However, the greatest challenge, the accurate determination of sperm morphology, should first be overcome. Following this, these systems will develop the potential to become a common alternative to, or even replacement for, current conventional sperm analysis methods. In conclusion, in today’s context, the novel smartphone-applied semen analyzer SpermCell™ represents a useful tool, with acceptable diagnostic accuracy, sensitivity, and specificity when used in determining the major semen parameters. It can be used as an efficient at-home point-of-care testing method in the initial assessment of couples with infertility concerns.

## Figures and Tables

**Table 1:** Comparison of the semen parameters between the standard manual semen analysis method and the SpermCell^TM^-based semen analysis method.

	Standard Manual Method	SpermCell^TM^-Based Method by Technician	SpermCell^TM^-Based Method by Patients	*p*
Sperm concentration (×10^6^/mL, Median ± IQR)	49.00 ± 64.50	54.20 ± 45.42	55.09 ± 47.33	0.90
Total Motile sperm count (×10^6^, Median ± IQR)	25.06 ± 37.06	23.34 ± 43.42	23.53 ± 41.50	0.46
Motile sperm percentage (%, Median ± IQR)	50.00 ± 20.00	47.40 ± 40.69	46.48 ± 40.09	0.43

**Table 2:** Sensitivity and specificity of SpermCell^TM^-based semen analysis administered by technicians for the diagnosis of oligospermia.

	Men with Oligospermia, n	Men with Normospermia, n	Total, n
Men with oligospermia in SpermCell^TM^-based analysis performed by the technician, n	9	2	11
Men with normospermia in SpermCell^TM^-based analysis administered by the technician, n	6	85	91
Total, n	15	87	102

Oligospermia: sperm concentration < 15 × 10^6^/mL via the standard semen analysis. Normospermia: sperm concentration ≥ 15 × 10^6^/mL via standard semen analysis. Sensitivity: 9/15, 60.00%. Specificity: 85/87, 97.70%.

**Table 3:** Sensitivity and specificity of the SpermCell^TM^-based semen analysis performed by patients in the diagnosis of oligospermia.

	Men with Oligospermia, n	Men with Normospermia, n	Total, n
Men with oligospermia in SpermCell^TM^-based analysis by administered by the patient, n	9	4	13
Men with normospermia in SpermCell^TM^-based analysis administered by the patient, n	6	83	89
Total, n	15	87	102

Oligospermia: sperm concentration < 15 × 10^6^/mL via standard semen analysis. Normospermia: sperm concentration ≥ 15 × 10^6^/mL via standard semen analysis. Sensitivity: 9/15, 60.00%. Specificity: 83/87, 95.40%.

**Table 4:** Sensitivity and specificity of the SpermCell^TM^-based semen analysis administered by technicians in the diagnosis of asthenozoospermia.

	Men with Asthenozoospermia, n	Men with Normospermia, n	Total, n
Men with asthenozoospermia in SpermCell^TM^-based analysis administered by technician, n	21	20	41
Men with normospermia in SpermCell^TM^-based analysis administered by technician, n	2	59	61
Total, n	23	79	102

Asthenozoospermia: motile sperm percentage < 40% via standard semen analysis. Normospermia: motile sperm percentage ≥ 40% via standard semen analysis. Sensitivity: 21/23, 91.13%. Specificity: 59/79, 74.68%.

**Table 5:** Sensitivity and specificity of the SpermCell^TM^-based semen analysis administered by the patients in the diagnosis of asthenozoospermia.

	Men with Asthenozoospermia, n	Men with Normospermia, n	Total, n
Men with asthenozoospermia in SpermCell^TM^-based analysis administered by the patient, n	21	18	39
Men with normospermia in SpermCell^TM^-based analysis administered by patients, n	2	61	63
Total, n	23	79	102

Asthenozoospermia: motile sperm percentage < 40% via standard semen analysis. Normospermia: motile sperm percentage ≥ 40% via standard semen analysis. Sensitivity: 21/23, 91.13%. Specificity: 61/79, 77.21%.

## Data Availability

The data presented in this study are available on request from the corresponding author. The data are not publicly available due to privacy or ethical restrictions.

## Supplementary Materials

The following supporting information can be downloaded at: https://www.mdpi.com/article/10.3390/diagnostics14070689/s1, Video S1: Video image of motile sperms; Video S2: Video image of non-motile sperms.
